# Mississippi River Plume Enriches Microbial Diversity in the Northern Gulf of Mexico

**DOI:** 10.3389/fmicb.2016.01048

**Published:** 2016-07-07

**Authors:** Olivia U. Mason, Erin J. Canter, Lauren E. Gillies, Taylor K. Paisie, Brian J. Roberts

**Affiliations:** ^1^Department of Earth, Ocean and Atmospheric Science, Florida State University, TallahasseeFL, USA; ^2^Louisiana Universities Marine Consortium, ChauvinLA, USA

**Keywords:** Mississippi River, Gulf of Mexico, ITag, microbial ecology, microbial diversity, bacterioplankton community composition, 16S rRNA gene sequencing

## Abstract

The Mississippi River (MR) serves as the primary source of freshwater and nutrients to the northern Gulf of Mexico (nGOM). Whether this input of freshwater also enriches microbial diversity as the MR plume migrates and mixes with the nGOM serves as the central question addressed herein. Specifically, in this study physicochemical properties and planktonic microbial community composition and diversity was determined using iTag sequencing of 16S rRNA genes in 23 samples collected along a salinity (and nutrient) gradient from the mouth of the MR, in the MR plume, in the canyon, at the Deepwater Horizon wellhead and out to the loop current. Analysis of these datasets revealed that the MR influenced microbial diversity as far offshore as the Deepwater Horizon wellhead. The MR had the highest microbial diversity, which decreased with increasing salinity. MR bacterioplankton communities were distinct compared to the nGOM, particularly in the surface where Actinobacteria and Proteobacteria dominated, while the deeper MR was also enriched in Thaumarchaeota. Statistical analyses revealed that nutrients input by the MR, along with salinity and depth, were the primary drivers in structuring the microbial communities. These results suggested that the reduced salinity, nutrient enriched MR plume could act as a seed bank for microbial diversity as it mixes with the nGOM. Whether introduced microorganisms are active at higher salinities than freshwater would determine if this seed bank for microbial diversity is ecologically significant. Alternatively, microorganisms that are physiologically restricted to freshwater habitats that are entrained in the plume could be used as tracers for freshwater input to the marine environment.

## Introduction

Over a decade ago the ubiquity of specific microbial lineages inhabiting global freshwater environments was reported (Zwart et al., 2002). More recently Newton et al. (2011) provided an extensive synthesis regarding what is known about freshwater microorganisms, including detailed phylogenies of freshwater clades, such as actinobacterial subclades that have been reported as one of the dominant phyla in lakes (Glockner et al., 2000; Warnecke et al., 2005; Allgaier and Grossart, 2006; Buck et al., 2009). In aquatic environments distinct microbial communities have been identified in freshwater as compared to marine environments (Rappé et al., 2000; Zwart et al., 2003). In comparing microbial diversity along a salinity trajectory from freshwater to the ocean, rivers have been implicated in enriching archaeal and bacterial diversity in coastal waters. Evidence of such an enrichment has been reported in the Columbia River estuary (Crump et al., 1999; Crump and Baross, 2000). Fortunato et al. (2012) showed that salinity and depth were the primary drivers structuring microbial communities, with microbial diversity decreasing from a riverine freshwater end member out to the shelf bottom in the Columbia River coastal margin. Fortunato and Crump (2015) similarly reported changes in microbial taxonomic composition along a salinity gradient profiled in three stations from the Columbia River to the Pacific Ocean. They also reported that microbial activity varied, but not systematically, with changes in salinity. In the Amazon River, metagenomic analysis of samples collected from the upper course of the river revealed that genes encoding heterotrophic processes were more abundant in comparison to those in the marine environment (Ghai et al., 2011). Satinsky et al. (2015) reported an increase in transcript copy compared to gene copy in five stations collected from the Amazon River to the ocean and suggested a greater per cell activity along this trajectory. These studies reveal that microbial community structure and function varies along a river to open ocean transect. They also show that microbial communities in river systems are distinct from high salinity ocean end members, with river systems often exhibiting higher diversity than open ocean sites. That rivers generally host a more diverse microbial community than marine environments suggests that river plumes, with reduced salinities and higher nutrient concentrations compared to the open ocean, may act as a seed bank for microbial diversity to marine waters as the plume migrates away from the river mouth and mixes with seawater. Whether freshwater microorganisms are active in river plumes as they migrate and mix with seawater would determine the ecological significance of this seed bank.

In the nGOM, the Mississippi (and Atchafalaya) Rivers are the primary sources of fresh water and key nutrients, delivering 80% of the freshwater inflow, 91% of the estimated annual nitrogen load, and 88% of the phosphorus load (Dunn, 1996). The freshwater, sediments, and dissolved and particulate materials are carried predominantly westward along the Louisiana/Texas inner to mid-continental shelf, especially during peak spring discharge (Rabalais et al., 2007). The influence of nutrient rich freshwater, in combination with stratification that arises from salinity differences and intensifies during summer with thermal warming of surface waters (Wiseman et al., 1997; Rabalais et al., 2010), is best exemplified by the formation of the annual, mid-summer hypoxic water mass that is distributed across the Louisiana shelf west of the MR and onto the upper Texas coast (Rabalais and Turner, 2001; Rabalais et al., 2007). However, MR plume transport is influenced by several factors such as wind, topography, interactions with boundary currents and eddies which can lead to offshore transport of the plume (Schiller et al., 2011 and references therein). This offshore movement of the low salinity, nutrient rich MR plume could influence the *in situ* microbial community structure and ecology beyond the historical area of low oxygen that occurs annually by acting as a dispersion mechanism for microbial diversity and conduit for introducing freshwater microorganisms, as it does with nutrients to the nGOM.

**FIGURE 1:**
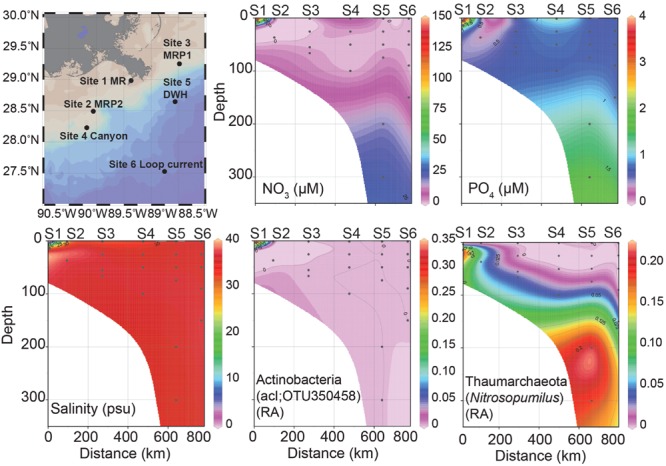
**Station map of six sites sampled, nutrient concentrations (NO_3_^-^, PO_4_^3-^), salinity (practical salinity units, psu) and relative abundances (RA) of Actinobacteria; acI and Thaumarchaeota; *Nitrosopumilus* profiles over depth.** In all figures distance is zero at the MR site and then proceeds: MRP2, MRP1, canyon, DWH to the loop current site at 800 km. These sites are plotted along a surface sample salinity trajectory from lowest (MR) to highest (LC). The sites are: Site 1 MR (S1), Site 2 MRP2 (S2), Site 3 MRP1 (S3), Site 4 canyon (S4), Site 5 DWH (S5), and Site 6 loop current (S6).

King et al. (2013) analyzed the microbial community in the MR to the open ocean in the nGOM. In their shallow, low salinity MR plume sample Proteobacteria were abundant, as were unclassified Bacteria, unclassified microbes and Verrucomicrobia. Although these authors reported that microbial diversity did not differ when comparing MR to marine samples, they did show that the MR sample had a distinct microbial community (King et al., 2013 see **Figure 1**). The distinct nature of their MR sample as compared to marine sites is likely influenced by the resident microbial community in the MR. For example, the Upper MR was dominated by Proteobacteria, Actinobacteria, Bacteroidetes, Cyanobacteria, and Verrucomicrobia (Staley et al., 2013), some of which overlapped with the major players in the MR plume as reported by King et al. (2013).

As shown by King et al. (2013) the MR did not harbor greater microbial diversity than nGOM seawater, as compared to the Columbia River which enriches microbial diversity in seawater as these salinity end members mix (Crump et al., 1999; Crump and Baross, 2000). Here we sought to further resolve whether the similarity in microbial diversity in the MR and the nGOM as reported by King et al. (2013) is a transient or stable feature by sampling the mouth of the MR to the loop current in the nGOM during the summer right before the onset of hypoxia, compared to King et al. (2013) who sampled in the spring. Specifically, we examined how the MR plume influenced microbial diversity from the mouth of the MR, in the MR plume, in the canyon, to the Deepwater Horizon wellhead and out to the loop current. To do so, we used iTag sequencing of 16S rRNA genes to characterize the microbial communities in 23 samples. We then analyzed this data along with *in situ* geochemistry and physical properties of the water column to determine the organizing principles structuring microbial communities along a salinity (and depth) gradient from a river end-member to the mesopelagic ocean.

## Materials and Methods

### Sample Collection

A total of six different sites were sampled during the CARTHE III pelagic cruise in the nGOM from July 7-10th, 2014 aboard the R/V *Pelican* (**Figure 1**). These six sites were the MI River mouth Southwest Pass (MR-P1; abbreviated as MR), MI River plume one (MRP1-P1; abbreviated as MRP1), MI River plume two (MRP2-S7; abbreviated as MRP2), loop current (LC-P4; abbreviated as LC), MI canyon (C-P5; abbreviated as C), and Deepwater Horizon wellhead (DWH-P3; abbreviated as DWH) (**Figure 1**). In total 23 samples were obtained. Sample nomenclature indicates the sampling location as well as the collection depth, which follows an underscore (e.g., MR-P1_3). Station MR-P1 bottom depth was 19 m. Collection depths were 1 m to 300 m, depending on the site. Physical properties including temperature, depth, pressure, and salinity (conductivity) were determined *in situ* using a Conductivity-Temperature-Depth (CTD) instrument.

### Oxygen and Nutrients

From one niskin bottle, duplicate water samples for dissolved inorganic nutrients (NO_3_^-^ + NO_2_^-^, NO_2_^-^, PO_4_^3-^, SiO_2_, and NH_4_^+^) were analyzed in triplicate using a Lachat Instruments QuikChem^®^ FIA+ 8000 Series Automated Ion Analyzer with an ASX-400 Series XYZ autosampler after being filtered through acid-cleaned (10% HCl) 47 mm diameter, 0.2 μm pore size, membrane filters (Pall Supor^®^-200) under low vacuum pressure. Samples were analyzed simultaneously for dissolved NO_3_ + NO_2_^-^ (by Cu-Cd reduction followed by azo dye colorimetry), PO_4_^3-^ (by the automated ascorbic acid reduction method), and SiO_2_^-^, but were analyzed separately for dissolved NH_4_^+^ (by phenate colorimetry) to prevent contamination of the samples by fumes from the NH_4_Cl buffer used in the analysis for NO_3_^-^ + NO_2_^-^ (APHA, 1992). Dissolved NO_2_^-^ was determined separately by azo dye colorimetry (without Cu-Cd reduction) and NO_3_^-^ concentration was determined by difference. Known volumes of water were filtered under low vacuum pressure onto precombusted (500°C for 4 h) 25 mm glass fiber filters (Whatman GF/F) and stored frozen prior to determination of particulate phosphorus concentrations using a modification of the method of (Aspila et al., 1976). Filters were placed in acid-cleaned (10% HCl) borosilicate vials and combusted at 550°C for 2 h. After cooling, 10 mL of 1N HCl was added to each vial and the vials were shaken for 16 h at 250-300 rpm. After settling, the samples were diluted 10-100 times and PO_4_-P was quantified using the automated ascorbic acid reduction method on the Lachat as described above.

Dissolved oxygen concentrations were determined at all locations and depths using the CTD oxygen sensor. At five stations and multiple depths (total *n* = 15) one or two (*n* = 4 occasions) 300-mL biological oxygen demand (BOD) bottles were filled from the same Niskin bottle using tygon tubing inserted into the bottom of a bottle and then allowing water to overflow the bottle by 2–3 volumes, taking care to expel all bubbles from the sample before capping. The bottles were immediately fixed (with 1 mL each of manganous sulfate and alkaline iodide solutions), stoppered, had deionized water added to fill the flared top and a secondary plastic cap to ensure a tight seal, and stored in the dark prior to titrations. Dissolved oxygen concentrations were determined using a modification of the Winkler titration method detailed in Strickland and Parsons (1972). Titrations were performed on three to five 50-mL aliquots per bottle using a Mettler Toledo DL28 auto-titrator. The mean and median coefficient of variation in DO concentrations among duplicate BOD bottles filled from the same Niskin bottle was 1.02 and 0.74%, respectively. Dissolved oxygen concentrations measured via winkler titration (95–237 μmol O_2_ L^-1^) spanned most of the range of DO concentrations observed in any profile during the sampling campaign. Ambient DO concentrations measured by winkler titration (DO_winkler_) were not significantly different than CTD measurements of DO (DO_CTD_) (*p* = 0.60; paired *t*-test) with the mean DO_winkler_ – DO_CTD_ of -0.69 mmol O_2_ m^-3^ (range: -5.7 to +6.8 mmol m-^3^) and a mean (DO_winkler_ – DO_CTD_)/DO_winkler_ value of -0.5%, providing a high degree of confidence in the DO patterns observed in CTD profiles.

### Microbial Sample Collection and DNA Extractions

At six stations 6-12 L of seawater was collected and filtered with a peristaltic pump. A 2.7 μm Whatman GF/D pre-filter was used and samples were concentrated on 0.22 μm Sterivex filters (EMD Millipore, Billerica, MA, USA). Sterivex filters were sparged and filled with RNAlater. DNA was extracted directly off of the filter by placing half of the Sterivex filter in a Lysing matrix E (LME) glass/zirconia/silica beads Tube (MP Biomedicals, Santa Ana, CA, USA) using the protocol described in Gillies et al. (2015) which combines phenol:chloroform:isoamyalcohol (25:24:1) and bead beating. Genomic DNA was stored at -80°C until purified. DNA was purified using a QIAGEN (Valencia, CA, USA) AllPrep DNA/RNA Kit. DNA quantity was determined using a Qubit2.0 Fluorometer (Life Technologies, Grand Island, NY, USA).

### 16S rRNA Gene Sequencing (iTag) and Analysis

16S rRNA genes were amplified from ∼10 ng of purified DNA in duplicate using primers 515F and 806R that amplify both bacteria and archaea, targeting the V4 region of *E. coli* in accordance with the protocol described by Caporaso et al. (2011, 2012) and used by the Earth Microbiome Project^1^, with a slight modification, specifically, the annealing temperature was modified from 50°C to 60°C. PCR amplicons were purified using Agencourt AMPure XP PCR Purification beads (Beckman Coulter, Indianapolis, IN, USA). Sequencing was carried out using the MiSeq (Illumina, San Diego, CA, USA) platform. Sequences were analyzed using QIIME version 1.9.0 (Caporaso et al., 2010) pipeline. Paired end reads were joined using fastq-join (Aronesty, 2011). Sequences were then demultiplexed and quality filtered using QIIME version 1.9.0 default parameters. These sequences are available at http://mason.eoas.fsu.edu and from NCBI’s sequence read archive (accession SRP077603). Sequences were then clustered into operational taxonomic units (OTU)s which was defined as ≥97% 16S rRNA gene sequence similarity with the open reference clustering protocol ^2^ with Greengenes version 13.5 (McDonald et al., 2012). The resulting OTU table was filtered to keep only OTUs that had at least 10 observations (6490 OTUs in total). Data was normalized using cumulative-sum scaling (Paulson et al., 2013). For the actinobacterial OTU350458 all 16S rRNA gene sequences from all samples that were 97% or more similar and thus grouped within this OTU were extracted and analyzed further by blastn. Specifically, using blastn the 350458 OTU representative 16S rRNA gene sequence was compared to all of the 16S rRNA gene sequences (55,163 sequences) in this OTU to examine phylogenetic cohesion.

### Statistics

Nutrient data were interpolated using Ocean Data View (Schlitzer, 2013). Alpha diversity (Shannon index) was determined according to Seaby and Henderson (2006) in QIIME (the QIIME script uses a default logarithm of 2 instead of *e*). The normalized OTU abundances in the 23 different samples were then analyzed using non-metric multidimensional (NMDS) scaling in *R* using metaMDS with default parameters in the Vegan package. To fit environmental vectors onto the ordination the Vegan function envfit was used. *P*-values were derived from 999 permutations of this data. A bipartite network of the 16S rRNA gene data was generated using QIIME. The network was visualized using Cytoscape’s edge-weight spring-embedded algorithm (edges were weighted by the abundance of an observation).

## Results and Discussion

### Chemical and Physical Properties of Samples

At 1.47 practical salinity units (psu), the MR sample collected at 3 m depth (MR-P1_3) serves as the low salinity, freshwater end-member (**Figure 1**; **Table 1**). At this same station, the 15 m MR sample salinity was 34.91 psu (**Figure 1**; **Table 1**) indicating penetration of nGOM water [using 36 psu as the reference salinity (Morey, 2003; Rong et al., 2014)] below the pycnocline in the river. The MR plume samples (MRP1and MRP2) had lower salinities in the surface (31.79 and 29.46 psu, respectively), increasing with depth (**Figure 1**; **Table 1**). The canyon and DWH samples had a similar salinity profile with surface sample salinities of 34.07 and 35.89 psu, respectively (**Figure 1**; **Table 1**). The loop current was the farthest from the MR and did not show a surface salinity dip compared to deeper depths, as observed at all other sampling locations.

**Table 1:** Metadata and physical data for MR, MRP, canyon, Deepwater Horizon wellhead, and loop current samples.

Sample name	Station	Depth (m)	Date sampled	Latitude	Longitude	Salinity (PSU)	Temperature (C)	Density(σ_𝜃_ (kg/m^3^))	Fluorescence (RFU)	Pressure (dbar)
MR-P1_3	River	3.3	7/7/14	28.987	-89.367	1.47	28.49	-2.80	0.71	3.28
MR-P1_15	River	14.5	7/7/14	28.99	-89.364	34.91	25.37	23.16	0.24	14.60
MRP1-P2_1	Plume 1	1.1	7/7/14	29.255	-88.478	31.79	29.56	19.47	1.20	1.09
MRP1-P2_25	Plume 1	25.4	7/7/14	29.255	-88.478	36.29	23.31	24.82	0.12	25.54
MRP1-P2_55	Plume 1	55.4	7/7/14	29.255	-88.478	36.39	20.63	25.66	0.20	55.83
MRP1-P2_66	Plume 1	66	7/7/14	29.255	-88.478	36.40	20.11	25.80	0.27	66.45
MRP2-S7_1	Plume 2	1	7/10/14	28.497	-90.054	29.46	30.11	17.54	0.24	1.00
MRP2-S7_37	Plume 2	36.5	7/10/14	28.495	-90.055	36.30	22.16	25.16	0.38	36.70
C-P5_1	Canyon	1.3	7/10/14	28.233	-90.174	34.07	29.14	21.32	0.04	1.29
C-P5_25	Canyon	25.2	7/10/14	28.233	-90.174	35.61	27.55	23.00	0.05	25.36
C-P5_50	Canyon	50.1	7/10/14	28.233	-90.173	36.31	21.59	25.33	0.13	50.49
C-P5_100	Canyon	99.7	7/10/14	28.233	-90.173	36.38	18.59	26.18	0.09	100.35
DWH-P3_1	Wellhead	1.2	7/8/14	28.65	-88.561	35.89	28.89	22.77	0.04	1.25
DWH-P3_25	Wellhead	25.4	7/8/14	28.65	-88.561	36.07	27.67	23.31	0.04	25.58
DWH-P3_50	Wellhead	49.7	7/8/14	28.651	-88.561	36.28	23.18	24.86	0.08	50.09
DWH-P3_75	Wellhead	74.5	7/8/14	28.651	-88.561	36.44	20.93	25.62	0.27	74.99
DWH-P3_200	Wellhead	199.6	7/8/14	28.65	-88.558	35.97	15.02	26.72	0.02	201.09
DWH-P3_300	Wellhead	300.4	7/8/14	28.65	-88.558	35.56	12.55	26.93	0.02	302.62
LC-P4_1	Loop Current	1	7/9/14	27.532	-88.75	36.30	28.95	23.06	0.04	1.03
LC-P4_25	Loop Current	25	7/9/14	27.531	-88.75	36.30	28.95	23.06	0.05	25.20
LC-P4_50	Loop Current	50	7/9/14	27.531	-88.75	36.30	28.21	23.30	0.07	50.34
LC-P4_90	Loop Current	89.9	7/9/14	27.532	-88.75	36.37	26.64	23.87	0.26	90.55
LC-P4_150	Loop Current	150	7/9/14	27.532	-88.75	36.83	22.33	25.52	0.04	151.11

Spatial patterns in dissolved nutrients across the sampling region were consistent with a strong influence of high nutrient MR water on the receiving waters of its plume (**Figure 1**). Locations within the plume (lower salinity) were higher in dissolved inorganic nitrogen (DIN) and phosphate (**Figure 1**; **Table 2**). Below the pycnocline, nutrient concentrations tended to increase with depth, as has been reported in previous studies (Shiller and Joung, 2012; Rakowski et al., 2015).

**Table 2:** Chemical data and Shannon diversity for MR, MRP, canyon, Deepwater Horizon wellhead, and loop current samples.

Sample name	Station	NO_2_ + NO_3_ (μm)	NO_2_ (μm)	NO_3_ (μm)	NH_4_ (μm)	DIN (μm)	PO_4_ (μm)	SiO_2_ (μm)	O_2_ (mmol/m^3^)	Shannon diversity
MR-P1_3	River	130.46	0.16	130.29	1.31	131.76	3.93	24.87	5.85	6.93
MR-P1_15	River	13.30	1.56	11.75	2.56	15.86	1.52	15.72	3.25	7.26
MRP1-P2_1	Plume 1	1.80	0.38	1.42	1.53	3.33	0.68	5.21	7.46	5.90
MRP1-P2_25	Plume 1	1.67	0.29	1.39	3.98	5.65	0.74	5.07	7.28	7.03
MRP1-P2_55	Plume 1	1.06	0.36	0.70	3.61	4.67	0.71	1.51	5.89	7.09
MRP1-P2_66	Plume 1	3.01	0.50	2.51	3.71	6.72	0.71	1.87	5.96	7.24
MRP2-S7_1	Plume 2	0.51	0.38	0.13	1.65	2.16	0.26	4.00	6.72	6.15
MRP2-S7_37	Plume 2	1.14	0.50	0.64	0.06	1.20	0.35	5.14	5.12	6.88
C-P5_1	Canyon	10.30	0.34	9.96	3.03	13.34	1.21	2.66	6.59	5.83
C-P5_25	Canyon	1.91	0.52	1.39	3.10	5.01	0.83	1.24	6.72	5.63
C-P5_50	Canyon	1.20	0.42	0.78	3.78	4.98	0.82	0.76	7.12	5.94
C-P5_100	Canyon	0.99	0.33	0.66	1.25	2.23	0.75	1.30	4.46	7.21
DWH-P3_1	Wellhead	1.08	0.32	0.76	3.51	4.60	0.71	1.21	6.65	5.47
DWH-P3_25	Wellhead	0.96	0.35	0.60	2.49	3.45	0.71	1.28	6.90	5.70
DWH-P3_50	Wellhead	0.98	0.35	0.63	2.95	3.93	0.74	1.19	7.36	5.01
DWH-P3_75	Wellhead	3.48	0.45	3.03	3.37	6.85	0.74	1.62	6.24	6.78
DWH-P3_200	Wellhead	19.14	0.42	18.72	2.68	21.82	1.31	6.03	4.35	7.10
DWH-P3_300	Wellhead	25.29	0.34	24.95	2.46	27.75	1.61	10.86	3.92	7.07
LC-P4_1	Loop Current	0.93	0.37	0.56	4.28	5.21	0.74	0.69	6.48	5.19
LC-P4_25	Loop Current	0.84	0.30	0.54	3.33	4.16	0.72	1.25	6.49	5.21
LC-P4_50	Loop Current	1.05	0.35	0.71	3.20	4.26	0.72	2.68	6.62	4.91
LC-P4_90	Loop Current	1.29	0.33	0.96	4.88	6.17	0.74	1.24	6.17	6.94
LC-P4_150	Loop Current	5.79	0.36	5.43	3.38	9.17	0.84	1.68	4.93	7.93

### Alpha Diversity

Two patterns emerged when comparing Shannon diversity across sample sites: (1) the MR hosted the highest diversity, which decreased with increasing distance away from the MR and (2) outside of the MR diversity increased with depth. In regards to the first trend surface and near-surface sites of comparable depths (two samples/site) revealed Shannon diversity (H) was highest in the MR (*H* avg = 7.10 *SD* = ± 0.19) and MR plume samples MRP1 and MRP2 (*H* avg = 6.50 ± 0.66, 6.52 ± 0.42, respectively; **Table 2**). Moving away from the MR Shannon diversity decreased from the canyon samples (*H* avg = 5.73 ± 0.11), to the DWH site (*H* avg = 5.57 ± 0.13) and finally to the loop current samples, which had the lowest Shannon diversity (*H* avg = 5.20 ± 0.01) (**Table 2**). The decrease in microbial diversity with distance from the MR contrasts with the findings of King et al. (2013) who reported no differences in diversity based on geographic location. We note that (1) these authors collected their samples at a different time of year (March) than we did (July), when environmental conditions may have been different and (2) our sample locations differed, both of which likely influenced variability in the microbial diversity observed in our studies. However, our data did agree with that of Fortunato et al. (2012) who reported the highest diversity in the Columbia River, which decreased as freshwater and seawater mixed resulting in increasing salinity. Our findings suggested that the MR hosts a more diverse microbial community than the surface waters of the nGOM. It has long been known that the MR influences *in situ* chemistry, but our data suggested that it may also act as a seed bank for microbial diversity as it mixes with the surface nGOM. This is consistent with previous reports that suggested the Columbia River acted as a source for enriching microbial diversity (both archaeal and bacterial) when mixed with seawater in the Columbia River estuary (Crump et al., 1999; Crump and Baross, 2000). However, we acknowledge deconvoluting the cause for higher microbial diversity in the MR plume as compared to the non-plume loop current samples is challenging, e.g., that higher nutrient concentrations allowed marine microorganisms to proliferate, rather than the MR acting as a seed bank for diversity. To this end evaluating the surface samples (6 samples from 6 sites) revealed that the 3 m MR sample had 2084 unique OTUs (47% of all surface samples with 4401 total OTUs). The loop current surface sample, which had no salinity anomaly that would have suggested freshwater mixing with seawater, had 99 unique OTUs (2%). This suggested that the MR plume could have influenced microbial diversity in two ways: (1) higher nutrient concentrations promoted the growth of marine microorganisms and (2) the MR plume introduced non-marine microorganisms to the nGOM.

Deeper in the water column the trend of decreasing Shannon diversity with increasing distance from the MR continued with the MR plume sample MRP1 having higher diversity at 55 and 66 m (*H* = 7.09 and 7.24, respectively) than the canyon 50 m (*H* = 5.94), DWH 50 m (*H* = 5.01) and finally the loop current 50 m sample (*H* = 4.91) (**Table 2**). A secondary noticeable trend, discussed above, is that the highest diversity at each deep water site is observed below 75 m (**Table 2**), which was not in agreement with the findings of King et al. (2013), but was reported by Fortunato et al. (2012) in the Columbia River coastal margin.

### Beta Diversity

**FIGURE 2:**
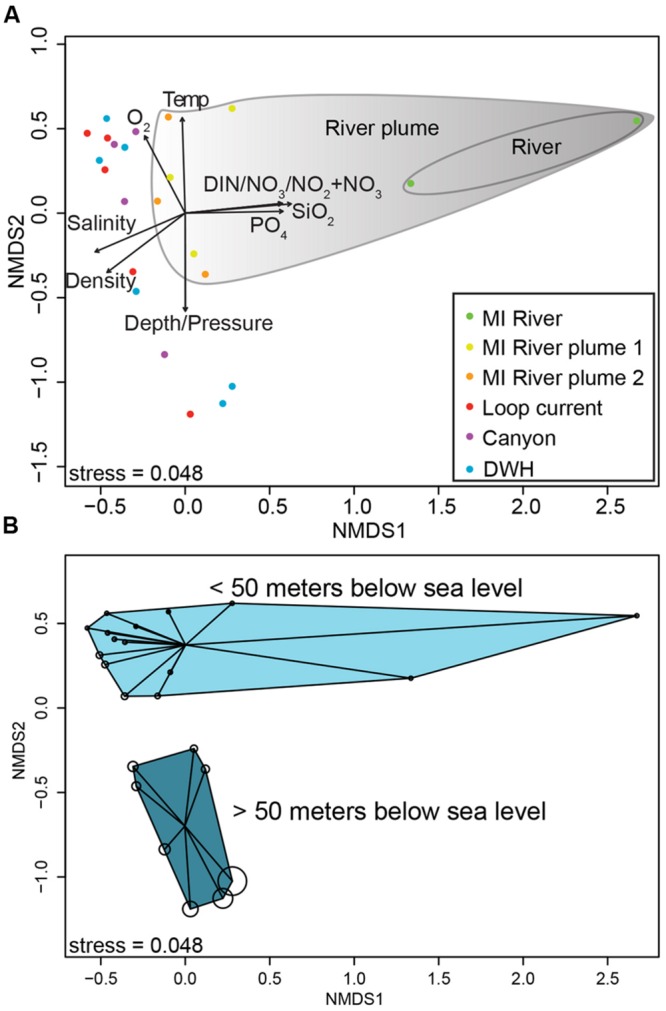
**Non-metric multidimensional scaling ordination plot of normalized 16S rRNA gene sequence data (A,B). (A)** Biplot showing correlations between environmental variables and ordination axes. **(B)** The same ordination, but shows depth by bubble size, which increases with increasing depth. **(B)** Shows sample clustering by depth (<50 m and >50 m).

The primary drivers in structuring the microbial communities were nutrient concentrations, salinity and depth (**Figure 2A**) as determined by non-metric multidimensional scaling ordination of normalized 16S rRNA gene data. In particular DIN, nitrate+nitrite, silicate, and phosphate were enriched in the MR, decreasing with distance from the mouth of the MR as shown by the vectors in **Figure 2** [these values were significantly correlated with an axis, with *p*-values ranging from 0.001 to 0.004 and correlation coefficients (*r*^2^) from 0.76 to 0.91]. Along this trajectory the microbial communities differed, particularly the shallow MR sample collected at 3 m depth (**Figure 2**). As expected salinity increased with distance from the MR (*p*-value = 0.006, *r*^2^= 0.77). Depth (*p*-value = 0.002, *r*^2^= 0.77) and the variables that change with depth (e.g., temperature and pressure) were also highly correlated with axes. Along this vertical trajectory the microbial communities formed shallow (0-50 m) and deeper (50-300 m) water column clusters (**Figure 2B**). The results of our beta diversity analysis are similar to those of Fortunato et al. (2012; see their **Figure 2**) where both salinity and depth were important drivers in structuring the microbial communities from the Columbia River out to the Pacific Ocean.

### Network Analysis

**FIGURE 3:**
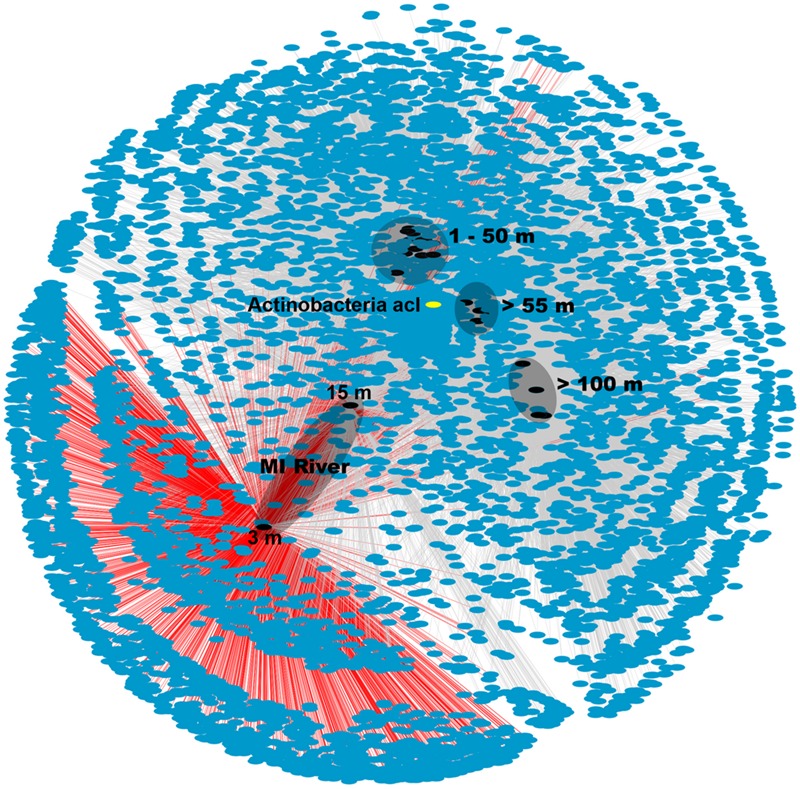
**Network analysis of OTU data from 16S rRNA gene sequences.** Sample nodes are black, OTU (7,111) nodes are blue and edges are gray. OTUs from MR-P1_3 m are connected via red edges. The different depths are indicated on the figure. The depths of the two MR samples are shown in the figure. The actinobacterial, acI OTU that was particularly prevalent in the shallow MR sample, node is shown in yellow.

The MR samples, and in particular the shallow 3 m sample, hosted a unique microbial community with many OTUs found only in this sample as shown in the network analysis figure (**Figure 3**). The number of unique OTUs observed in this sample (2,019 or 31% of all OTUs) agreed with the high Shannon diversity, relative to the other samples, discussed above. While there were also unique OTUs (782 or 12%) in the deeper MR sample (15 m) most were shared with its shallow MR counterpart or with nGOM seawater samples (**Figure 3**). The pattern of shared OTUs from the freshwater MR end-member to nGOM marine samples in this sample was expected given the observed mixing of freshwater with nGOM seawater at this sampling location as indicated by the salinity. Similar to the ordination (**Figure 2**) the shallow MR plume samples clustered together and with nGOM samples collected in the near surface (≤50 m) of the water column (**Figure 3**). Finally, the remaining samples clustered by depth (**Figures 2** and **3**). As observed in the ordination (**Figure 2**), samples clustered by nutrient concentrations, salinity and depth (**Figure 3**). Further, the pattern of unique and shared OTUs in the shallow MR sample to unique and shared OTUs in the deeper MR sample further supported that the MR acts as a microbial seed bank, introducing microbial diversity to the nGOM as freshwater and seawater mix.

### Microbial Community Structure in the Mississippi River, Plume and Northern Gulf of Mexico

**FIGURE 4:**
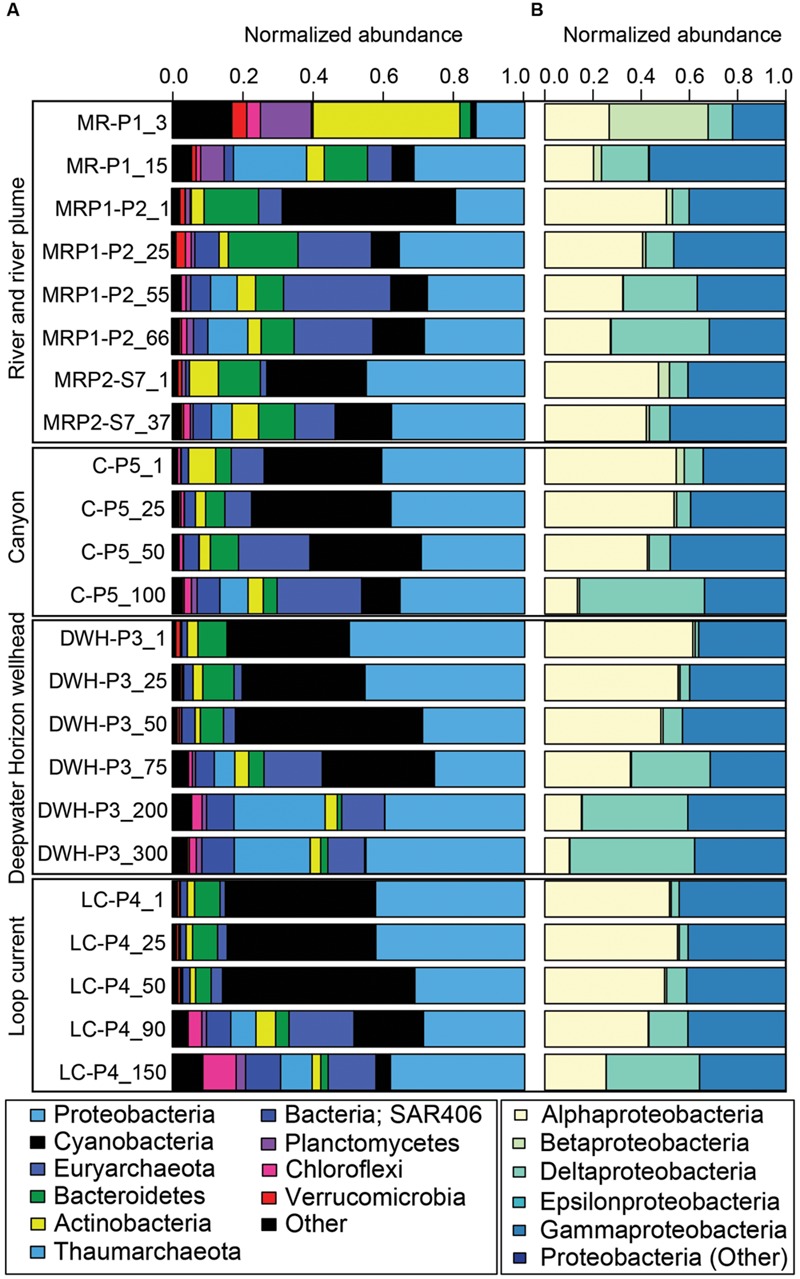
**Bar graph of normalized 16S rRNA gene sequence data. (A)** Shows the relative abundance of the most abundant phyla. Less abundant phyla are grouped under “Other.” The figure key shows the highest (Proteobacteria) to lowest abundances (Other). **(B)** Shows relative abundance of the different proteobacterial sub-phyla. Less abundant proteobacterial sub-phyla are grouped under “Other.” The figure key shows the highest (Alphaproteobacteria) to lowest abundances (Proteobacteria; Other).

The data revealed that the 3 m MR sample (MR-P1_3 m) had a highly divergent microbial community compared to the other samples (**Figures 2–4**). Specifically, this high nutrient, low salinity end member sample collected in the mouth of the MR was dominated by the Actinobacteria phylum (42% of all phyla in MR-P1_3 m/avg. 5% ± 3% in non-MR surface samples collected from 1 m) and to a lesser degree Proteobacteria and Planctomycetes (both were 14%/avg. 39% ± 12% and 0.6% ± 0.6%), Verrucomicrobia (4%/avg. 0.9% ± 0.5%), Chloroflexi (4%/avg. 0.2% ± 4%), and unclassified microorganisms (17%/avg. 2% ± 0.5%) (**Figure 4**). This shallow MR sample had lower relative abundances of Cyanobacteria (1%/avg. 38% ± 8%), Bacteroidetes (3%/avg. 9% ± 4%), and Marine Group II Euryarchaeota (MGII) (0.01%/avg. 3% ± 3%) (**Figure 4**). The relative abundances of the other phyla presented in **Figure 4** were similar in these surface samples, or were less than 1% in relative abundance and are not discussed in more detail here. In the 3 m MR sample relative betaproteobacterial abundance was high (41% of all proteobacterial subphyla), followed by Alphaproteobacteria (27%), Gammaproteobacteria (22%), and Deltaproteobacteria (10%) (**Figure 4B**). King et al. (2013) reported MR surface water had high abundances of unassigned bacteria (their **Figure 4A**) and Betaproteobacteria. In contrast, our non-MR surface samples had low betaproteobacterial abundances ranging from less than 1 to 4.5% of the Proteobacteria with Alphaproteobacteria being most abundant (47 to 61%) followed by Gammaproteobacteria (34 to 44%) (**Figure 4B**). The dominance of Proteobacteria, and in particular Alphaproteobacteria in our shallow nGOM samples is consistent with the findings of King et al. (2013). Our surface plume, canyon, loop current and DWH samples also had high normalized abundances of Cyanobacteria (28-49% of all phyla, compared to 1%), which were excluded from the analysis presented by King et al. (2013). In contrast to our shallow MR sample, Bacteroidetes were well represented in our surface nGOM samples (4-15% of all phyla compared to 3%), consistent with King et al. (2013).

In the deeper MR sample (MR-P1_15 m) the microbial community was less divergent compared to the other sites than the shallow 3 m MR sample (**Figures 2–4**). This greater congruency of the MR-P1_15 m sample with marine samples collected from 25 to 50 m is likely due to mixing of MR and seawater as indicated by a salinity of 34.91 psu. Specifically, in the 15 m MR sample, Proteobacteria were most abundant (31% of all phyla) with Gammaproteobacteria accounting for 56% of the Proteobacteria (avg. 43% ± 4% in non-MR samples collected from 25 to 50 m) while Alphaproteobacteria and Deltaproteobacteria were 20%/avg. 48% ± 6% and avg. 7% ± 3%. Betaproteobacteria were not abundant in MR_15 m (3% of Proteobacteria) compared to its shallow counterpart (41% of Proteobacteria), which is more congruent with non-MR samples in the <50 m depth interval, in which Betaproteobacteria abundances were low (avg. 0.9% ± 0.2% of all Proteobacteria). Cyanobacteria abundances were low (6% of all phyla) in the 15 m MR sample as compared to the non-MR sites (avg. 35% ± 16%). The deeper MR sample had high relative Thaumarchaeota abundances compared to other samples in this depth interval (21%/ avg. 0.7% ± 2%). The high relative abundance of Thaumarchaeota in the MR was not a feature of the shallow MR sample analyzed in King et al. (2013), and, to our knowledge is the first observation that Thaumarchaeota are abundant in the MR. The relative abundance of MGII was low in the shallow MR sample (less than 0.01%), increased to 6% in the 15 m sample, but was slightly lower than the <50 m non-MR samples at avg. 7% ± 6%. The relative abundance of Bacteroidetes was 12% in the 15 m MR sample, compared to 9% ± 5% in non-MR samples. Finally, the MR_15 m differed from the other samples in that it had higher relative abundances of Planctomycetes (7% compared to avg. 0.3% ± 0.3%), which was not a feature observed in the shallow MR sample presented by King et al. (2013).

### The Mississippi River as a Conduit for Introducing Microorganisms to the Northern Gulf of Mexico

#### Bacteria

The high microbial diversity in the MR and the elevated diversity in surface seawater influenced by the MR plume in this study suggested that the MR could in fact act as a seed bank for microbial diversity as it mixes with the nGOM. The difference in microbial community composition between the low salinity end member and marine samples was primarily due to the high relative abundance of Actinobacteria, which decreased with increasing distance from the MR. Thus a more detailed analysis of this group follows. Blastn analysis revealed that the most abundant actinobacterial OTU (OTU350458) averaged 99.5% similarity, 0.8 mismatches, 463.6 bit score and 1.2 × 10^20^ Expect value to the 16S rRNA gene sequences in this OTU (55,163 sequences in total). This additional analysis suggested that the representative OTU350458 captured only highly similar to identical 16S rRNA gene sequences in this OTU. OTU350458 was classified as ACK-M1 (Zwart et al., 2002), now referred to as acI (Warnecke et al., 2004). This single actinobacterial OTU (OTU350458) was observed in nearly all of the samples (**Figure 3**), but its relative abundance was highest in the 3 m MR sample (32% of the microbial community), decreased to 0.9% in the 15 m MR sampled and was low (< 0.01%) to undetectable outside of the MR. Further, in our dataset this acI OTU was inversely correlated with salinity (Spearman ρ = -0.47, *p*-value = 0.01). This actinobacterial OTU was most similar in 16S rRNA gene sequence to microorganisms from freshwater environments, but also 100% similar to several clones from the Baltic Sea in seawater collected below ice (e.g., Acc LM652066). Although OTU350458 was the most abundant acI up to 23 different acI OTUs were observed across the samples, with the highest diversity observed in the MR (3 m) sample (all 23 OTUs were present). All acI OTUs were significantly inversely correlated with depth (ρ = -0.67, *p*-value = < 0.0), salinity (ρ = -0.50, *p*-value = 0.01) and density (ρ = -0.60, *p*-value = < 0.0) and positively correlated with temperature (ρ = 0.51, *p*-value = 0.01).

Recently Newton et al. (2011) provided a comprehensive synthesis of freshwater microbial communities from lakes and reported that Actinobacteria was one of the five most numerically dominant phyla in lake epilimnia. This finding is consistent with an earlier synthesis of freshwater microbial communities by Zwart et al. (2002) who reported that specific clades of Actinobacteria were well represented in freshwater. Zwart et al. (2002) also revealed that Actinobacteria were found in estuaries and the coastal ocean, but not in the open ocean. Warnecke et al. (2004) delineated and described the acI as a freshwater actinobacterial cluster that is highly represented in lakes and rivers, and to a lesser degree in estuaries.

Although Actinobacteria in the acI clade are numerically dominant in freshwater ecosystems they have eluded cultivation efforts, thus their salinity optimum and activity along a salinity gradient is unknown. Further, the lack of cultivated representatives required methodologies that circumvent the need to culture to determine physiology. In this vain Garcia et al. (2012) used single cell genomics and described the organism SCGC AAA027-L06. This single cell was 95% similar to our acI OTU350458 (it should be noted that neither our, nor their 16S rRNA genes are full-length) and based on 16S rRNA gene sequence is the most similar single cell genome, inclusive of those presented in Ghylin et al. (2014). Using the acI representative sequences in Garcia et al. (2012) places the OTU350458 in the acI-C2 sub-clade (data not shown) while their SCGC AAA027-L06 is in the acI-B1 sub-clade. They reported that SCGC AAA027-L06 has a small genome that encodes a facultative aerobic lifestyle, with numerous enzymes involved in pentose utilization. Additionally, microautoradiography and fluorescence *in situ* hybridization (MAR-FISH) showed acI actively assimilated low-molecular-weight organic compounds, the source of which was suggested to be phytoplankton exudates (Salcher et al., 2013). These previous studies reveal a ubiquitous freshwater clade, representatives of which could degrade phytoplankton exudates.

The metabolism of acI led us to turn our attention to our other dataset (Gillies et al., 2015), namely the annual nGOM dead zone. In that dataset the same OTU (99% similar) was present and most abundant at the mouth of the MR in 2013, but was nearly absent moving westward over the shelf. We also evaluated its abundance in the 2014 hypoxic zone, where the hypoxic area was concentrated at the mouth of the MR, and found that it was abundant in the MR (29% relative abundance in the mouth) and in surface samples moving westward from the MR (Gillies et al., unpublished data). On this trajectory salinities ranged from 2 in the MR to 35.5 psu (avg. 22 psu), which is close to the seawater salinity value defined above. We hypothesize that acI is abundant in the MR, and during spring runoff it, along with excessive nutrients and reduced salinity, are introduced to the nGOM. During the resulting algal bloom if acI is metabolically active in the freshwater MR plume at salinities that exceed freshwater, but are below marine salinities, it would be well poised to rapidly degrade low molecular weight compounds, such as those found in algal exudates, and could even do so in low oxygen environments. Thus, we hypothesize that acI may play a role in establishing the hypoxic conditions that prevail near the mouth of the MR during the summer in the reduced salinity layer that overlays the saline nGOM bottom water. However, this hypothesis has not been tested herein, nor do we have the data to determine if the acI are metabolically active in the lower salinity MR plume, but the presence of some acI clade members in estuaries and in the Baltic Sea suggest that it may be active along a salinity gradient. Alternatively, if the acI is physiologically restricted to freshwater, its decreasing relative abundance outside of the mouth of the MR with increasing salinities and depths, along with early reports describing this clade as freshwater adapted, suggested that acI Actinobacteria may be a plausible tracer for freshwater input to the marine environment. We envision a quantitative assay for acI, such as the quantitative polymerase chain reaction that could theoretically provide a tracer for river input to seawater.

#### Archaea

Thaumarchaeota in the MR has not previously been reported (King et al., 2013), suggesting that at certain times during the year, deeper water in MR may host greater archaeal diversity than the shallow nGOM. In our samples, the majority of Thaumarchaeota were in the genus *Nitrosopumilus*, a marine microorganism, therefore we focus on the distribution of this genus from the MR to the loop current. The relative abundance of this genus was low in all surface samples, including the 3 m MR sample (although at <1% of all genera in this sample it was the highest of any surface sample) (**Figures 1** and **4**). The deeper MR sample (15 m) had high *Nitrosopumilus* abundances at 19% of all genera compared to 0.63% ± 2% in the other samples of comparable depths (25-50 m) (**Figures 1** and **4**). High relative abundances of Thaumarchaeota closely related to *Nitrosopumilus maritimus* (Könneke et al., 2005) have been reported in the 2013 nGOM hypoxic zone (Gillies et al., 2015). In the 2014 hypoxic zone *N. maritimus* was again highly abundant, as were *amoA* genes, particularly in or near the mouth of the MR, where salinity was lower than typical seawater (Gillies et al., unpublished). This data suggested that the deeper MR may introduce Thaumarchaeota closely related to *N. maritimus* to the shallow nGOM water column as it mixes and moves westward. In Gillies et al. (2015) we postulated that persistent archaeal hotspots that were predominantly *Nitrosopumilus* in the hypoxic area would serve as a site where energy flow is diverted from higher trophic levels to, in this case, ammonia-oxidizing Thaumarchaeota. This aerobic metabolism would result in sustained oxygen draw down in an oxygen-depleted environment. Thaumarchaeota, and *N. maritimus* in particular, are viable and abundant at seawater salinities. Thus their introduction to the nGOM from the deeper MR may be an example of the MR enriching seawater with ecologically significant microorganisms, however, different methodological approaches are required to test this hypothesis.

## Conclusion

In this study we used iTag sequencing of 16S rRNA genes for 23 samples collected in the MR, in the MR plume, in the canyon, at the Deepwater Horizon wellhead and out to the loop current, and *in situ* geochemistry to determine whether the MR influences microbial diversity in the nGOM. This analysis revealed that the MR had a distinct microbial community compared to marine samples and also had the highest diversity of any sample site. We suggest that the MR could in fact act as a seed bank for microbial diversity as it mixes with seawater in the nGOM. Future work will be directed at creating a quantitative assay to determine whether the freshwater acI actinobacterial clade could be used as a tracer for freshwater input to the marine environment. Additionally, we endeavor to determine if the acI clade and Thaumarchaeota that are introduced by the surface and near surface waters of the MR to the nGOM are ecologically significant.

## Author Contributions

OM conceived of the experiments, did the bioinformatics and statistical analyses of the data and wrote the manuscript. EC carried out DNA extractions, library preparation and sequencing. LG and TP participated in the cruise to obtain samples. BR determined nutrient and oxygen concentrations.

## Conflict of Interest Statement

The authors declare that the research was conducted in the absence of any commercial or financial relationships that could be construed as a potential conflict of interest.

## Footnotes

1 http://www.earthmicrobiome.org/emp-standard-protocols/16s/

2 http://qiime.org/scripts/pick_open_reference_otus.html
